# Exploring the emotional labour of occupational therapists when interacting with clients

**DOI:** 10.1177/03080226251363728

**Published:** 2025-08-30

**Authors:** Rebecca F Hings, Katarzyna Furmaniak, Carolyn Dunford, Christopher RD Wagstaff, Alessandro Quartiroli

**Affiliations:** 1 Department of Sport, Health and Exercise Sciences, Brunel University of London, UK; 2 Central and North West London NHS Foundation Trust, UK; 3 Department of Health Sciences, Brunel University of London, UK; 4 School of Sport, Health and Exercise Science, University of Portsmouth, Portsmouth, UK; 5 Department of Psychology, University of Wisconsin La Crosse, La Crosse, WI, USA

**Keywords:** Emotions, emotion management, therapeutic relationship, professional practice

## Abstract

**Introduction:**

Occupational therapy is an inherently emotional endeavour for both clients and therapists. Despite navigating diverse and challenging work environments, relatively little is known about how occupational therapists are expected to manage and express their emotions in line with job-related emotion norms. The aim of this study was to explore the emotional labour experiences of occupational therapists when interacting with clients.

**Method:**

Fourteen qualified occupational therapists (11 female and 3 male) took part in a semi-structured interview about practice-related emotion norms, emotion regulation and emotion performance demands. Data were analysed using reflexive thematic analysis.

**Findings:**

Two overarching themes captured the complexities of emotional labour when interacting with clients including (1) navigating subjective emotion norms, and, (2) negotiating authentic and inauthentic emotions to nurture the therapeutic relationship. Prioritizing client needs informed how and what emotions were expressed and regardless of the emotional labour strategies adopted, therapists strived for perceptions of sincerity in their relationships with clients.

**Discussion:**

The enactment of emotional labour when interacting with clients is critical to delivering therapy and meeting professional standards. The findings have implications for practitioners (e.g. reflective practice, self-care), managers (e.g. supervision) and organizations (e.g. support structures, education) and point to fruitful avenues for research.

Working with clients to deliver good-quality, person-centred healthcare presents a unique challenge for occupational therapists. For example, Kolehmainen and McAnuff (2014) found children’s occupational therapists experienced feelings of anger, frustration, guilt, inadequacy and worry when they felt unable to achieve positive outcomes with clients. The tensions between values that underpin professional practice (e.g. compassion, care, altruism; Thomas et al., 2019) and neo-liberal healthcare systems (e.g. cuts to government spending; Hayes et al., 2020) might lead to a potential imbalance in social relations between the practitioner and the client in terms of *how* occupational therapists are expected to manage emotions during social interactions. Therefore, practitioners are required to regulate emotions and expressions in order to fulfil interpersonal work-related expectations; a concept defined as emotional labour (Gabriel et al., 2023; Hochschild, 1983). For example, occupational therapists need to manage their emotions in order to develop appropriate therapeutic alliances with clients (Horton et al., 2021), implement person-centred approaches to service delivery (Wallengren et al., 2022), and facilitate productive working relationships with colleagues, managers and multidisciplinary teams (Bowser et al., 2025) in a variety of divergent working environments such as hospital wards, care homes, rehabilitation centres. Although enacting emotional labour can help support working relationships and manage conflicts with colleagues (Riley and Weiss, 2016), researchers have reported emotional labour to be an underappreciated yet critical pre-requisite to healthcare provision (Elliott, 2017). Indeed, the human costs of emotional labour such as emotional exhaustion, burnout, anxiety and depressive symptoms and even physical ill-health symptoms (Hülsheger and Schewe, 2011) might contribute to problematic practitioner turnover rates in occupational therapy (Hings et al., 2024). Despite the widely reported benefits and disadvantages of emotional labour in healthcare literature, relatively little attention has been afforded to the emotional labour of practicing occupational therapy.

The tri-focal theory of emotional labour (Grandey and Gabriel, 2015) suggests that emotional labour comprises three interconnected processes, namely, emotion requirements, emotion regulation and emotion performance. Emotion requirements, otherwise termed emotion norms, are job-related expectations for emotional displays which are often implicit in nature that govern how one enacts emotional labour in the workplace. For example, Kirk et al. (2021) found that Emergency Department nurses were expected to display a wide array of context-dependent emotions when interacting with patients including calmness, fearlessness and empathy. Such emotion norms are influenced by factors like professional codes of conduct, organizational structures, education, social networks, work-spaces and group power dynamics (McCloughen et al., 2020). To enact contextually appropriate emotions during a social interaction at work, three emotional labour strategies are typically adopted: (1) surface acting (i.e., faking one’s expressions by amplifying or suppressing emotional displays), (2) deep acting (i.e. purposefully changing how one feels to match the desired expression needed in the moment by means of cognitive reappraisal and behavioural strategies) and (3) authentic emotional expression (i.e., automatic regulation and expression of desired emotions). Based on how our emotions are regulated, emotions are then observably performed to others by alterations in body language, verbal expressions and facial expressions that are required for a particular situation. When surface acting or deep acting, the mismatch between one’s felt emotions and expressed emotions can lead to emotional dissonance and a sense of inauthenticity when interacting with others at work which can lead to decreased job satisfaction and higher levels of burnout (Hülsheger and Schewe, 2011).

In recent years, researchers have explored the influence of various emotion-related processes on the professional practice of occupational therapists. For example, the ability to interpret the emotions of yourself and others (i.e., emotional intelligence; Andonian, 2017), the deliberate use of emotion regulation to affect others’ responses (i.e. interpersonal emotion regulation; Horton et al., 2022), and the protective mechanisms required to cope with the emotional demands (i.e. resilience; Roundy et al., 2023) have been investigated in the occupational therapy context. Despite issues with conceptual overlap of such emotional phenomena (cf. Tamminen et al., 2022), the collective and emerging knowledge on such emotion-related processes has been evidenced to enhance practitioner self-awareness, maintain professional boundaries within therapeutic relationships, and protect the wellbeing of occupational therapists. To our knowledge, one study has acknowledged the influence of emotional labour on the practices of occupational therapy students on clinical placements (Healey, 2017). Adopting a post-structuralist perspective on emotions, Healey (2017) used creative arts-based methods with seven participants that highlighted the specific emotions felt, embodied and enacted by students on placement within the constraints of university (e.g. needing to pass placement modules and gain approval of placement educators) and workplace structures (e.g. obtaining favourable reports from other professionals who act as gatekeepers to the profession). Therefore, in this study, our aim was to significantly extend this work by seeking to understanding how *qualified* therapists with differing career trajectories experience emotion, interpret emotion norms, cultivate strategies for emotion regulation and expression when interacting with clients.

Occupational therapy work provides novel, varied and challenging contexts to extend theory on emotional labour. For example, depending on the role or organization, therapists are expected to develop rapport with clients in transient (e.g. accident and emergency visit) and/or longer-term therapeutic alliances (e.g. clients residing in residential units). Further, occupational therapists are, at times, required to negotiate potentially intimate interactions through physical touch (Klamroth-Marganska et al., 2021), provide personalized support for multiple mental and physical health conditions (Leland et al., 2017), enter private spaces such as clients’ homes (Robertson and Fitzgerald, 2010), and adopt person-centred care approaches (e.g. Wallengren et al., 2022) while maintaining a ‘professional’ outward countenance (Walder et al., 2022). In other healthcare domains, emotional labour has been shown to serve as a useful reflective tool for raising awareness of the influence of emotions during practice (Edward et al., 2017). Indeed, previous research has highlighted: (1) the importance of emotion-related processes in healthcare roles (Andonian, 2017), (2) the complex emotional demands occupational therapists encounter (Horton et al., 2022), (3) and the personal and professional costs associated with emotional labour (Riley and Weiss, 2016), Therefore, in this study, we aimed to explore the emotional labour experiences, including norms, emotion regulation and emotion performance, of occupational therapists when interacting with clients using a qualitative semi-structured interview design.

## Method

### Ethics

This project secured ethical approval from the [redacted] Ethics Committee (35997-MHR-Apr/2022- 39206-2). Each participant provided a priori written informed consent.

### Research design

For the purpose of this study, the researchers adopted a relativist ontological (i.e. reality is multiple and individually constructed via unique lived experience such as shared language and meanings) and social constructionist epistemological assumptions (i.e. knowledge is created via social interactions between individuals and the world; Hoffmann et al., 2022). Therefore, a qualitative descriptive methodology in the form of a cross-sectional semi-structured interview design was adopted. The main focus of the investigation was to provide a novel and detailed description of the emotional labour experienced by occupational therapists (Doyle et al., 2020).

### Participants and recruitment

Fourteen participants agreed to share their experiences for the study (see Table 1). Participants were recruited using purposive convenience sampling (Robinson, 2014). The study was advertised on the research team’s social media channels (e.g. X and Facebook) and via word of mouth to occupational therapists within our social and professional networks. To take part, participants were required to be aged 18 years and above, actively practicing as an occupational therapist in the United Kingdom in any capacity, and be registered with the Health Care and Professions Council (HCPC). Although some participants held substantive roles as university lecturers, participants had experience practicing in various areas of occupational therapy including mental health, forensics, rheumatology, hand therapy, dementia and paediatrics.

**Table 1.** Participant socio-demographic characteristics.

Participant pseudonym	Self-identified Gender (Pronouns)	Self-identified race/ethnicity	Years of experience (Range)	Current job position(s)
Michael	Male (he/him/his)	Chinese	25+ years	University Lecturer
Harley	Female (she/her/hers)	White/Other	5–10 years	Occupational Therapist (Band 6; NHS)
Leon	Male (he/him/his)	White/British	5–10 years	Senior Occupational Therapist (in Local Authority)
Martina	Female (she/her/hers)	White/Other	5–10 years	University Lecturer
Chuck	Male (he/him/his)	Mixed Heritage	5–10 years	Occupational Therapist (Band 6; NHS)
Rhia	Female (she/her/hers)	White/British	5–10 years	University Lecturer
Rayne	Female (she/her/hers)	White/British	5–10 years	Hourly Paid University Lecturer
Lizzy	Female (she/her/hers)	White/British	5–10 years	Senior Occupational Therapist (Band 7; NHS)
Nina	Female (she/her/hers)	Black/African	0–5 years	Occupational Therapist (Band 5; NHS)
Delphine	Female (she/her/hers)	Asian/Chinese/Hong Kong	0-5 years	Rotational Occupational Therapist (Band 5; NHS)
Cara	Female (she/her/hers)	White (Ethnicity Not Stated)	5–10 years	Band 6 Occupational Therapist (Band 6 and Band 7; NHS) and University Lecturer
Skye	Female (she/her/hers)	Asian/Other	0–5 years	Occupational Therapist (Band 5; NHS)
Millie	Female (she/her/hers)	White/Australian	5–10 years	Senior Occupational Therapist (Band 7; NHS)
Amira	Female (she/her/hers)	White/British	10–15 years	Clinical Specialist Occupational Therapist (Band 6; NHS)

NHS: National Health Service (United Kingdom). For further information about NHS salary bands and job responsibilities, please visit the following website: https://www.nhsbands.co.uk/ ‘Local Authority’ refers to a structure of local government in England such as a District or Borough/City Council (National Health Service, 2022).

### Data collection

Once participants gave their consent, a one-to-one semi-structured interview was scheduled at a mutually convenient time. Given the geographical spread of the participants, interviews were conducted either in-person in a private space (e.g. classroom on university campus) or online via Microsoft Teams or Zoom. Despite differences in interview mediums, online platforms increase accessibility (e.g. time and cost saving for participants) and maintain a personal interface to discuss sensitive topics. The interviews lasted an average of 85 minutes, totalling 19 hours and 54 minutes of audio. The interviews were transcribed verbatim using Microsoft Word and resulted in 763 pages of double-spaced typed text.

### Interview guide

Using theory on emotional labour (e.g. Grandey and Gabriel, 2015) as a guiding framework as an initial starting point to shape conversations, a semi-structured interview guide was created comprising three areas: (a) background, (b) emotional labour and (c) recommendations. Nevertheless, whilst this interview guide acted as a scaffold for conversations during the interview, in line with our philosophical positionality, we invited participants to be partners in the construction of knowledge. The first section was designed to enhance rapport with the participants by learning about their careers as well as pinpointing specific environments, contexts and interactions that might be critical to unpick during the interview (e.g. can you tell me about your occupational therapy career up to now?) The second section explored emotion norms (e.g. to what extent are there expectations about you should express your emotions as an occupational therapist?), emotion regulation (e.g. can you tell me about a situation where you had to hide your true feelings about a situation/issue/event in your role?) and emotion performance (e.g. during an interaction at work, how do you show your emotions to others?). The final section explored recommendations participants might have for managing emotional labour demands (e.g. what advice would you give to aspiring occupational therapists about dealing with emotions and emotional expressions in their role?). To support the interview guide, a list of probing questions was developed to support participants to expand on their experiences and to extend conversation relevant to the research aim (DeJonckheere and Vaughn, 2019).

### Data analysis and quality indicators

Data were analysed using a reflexive approach to thematic analysis (Braun and Clarke, 2021). An abductive reasoning approach was taken whereby the researchers’ moved flexibly between pre-existing knowledge (e.g. tri-focal theory of emotional labour; Grandey and Gabriel, 2015) and discovery of novel concepts that cannot be explained by existing knowledge (e.g. the Occupational Therapy context) when interpreting the dataset (Kennedy and Thornburg, 2018). Please see Table 2 for a description of data analysis procedures for this study.

**Table 2.** Phases of Reflexive Thematic Analysis (RTA) adopted in this study (Braun and Clarke, 2021).

RTA phase	Phase description	Application to study
(1) Data familiarization	Deep analytical immersion in the dataset by transcribing, reading and re-reading interview transcripts.	The first and second authors familiarised themselves with the interview transcripts by repeatedly reading the transcripts and recording initial impressions of the dataset.
(2) Data coding	A systematic labelling process of data relevant to the research aim in the form of (a) semantic codes (i.e., literal translations) and (b) latent codes (i.e., interpretive labels).	The first and second authors coded the interview transcripts on a line-by-line basis including written descriptions in relation to the research question.
(3) Theme development	Clustering and describing overarching patterns of codes into central organizing concepts (i.e., initial themes) relevant to the research aim.	The research team grouped specific codes into initial themes using emotional labour theory as a guiding framework.
(4) Theme refinement	Reflecting on whether the central organizing concepts require revision based on distinctiveness, relationships between themes, and whether the themes fit with the research aim. Thematic structuring in the form of overarching themes, themes and subthemes.	The research team held a meeting whereby the initial themes were presented, critiqued and rearranged.
(5) Theme naming	Developing thematic structure alongside theme titles, definitions and descriptions related to the research aim.	A full representation of the thematic structure including theme titles, definitions and descriptions were created.
(6) Writing up	Although integral to the RTA process, the final stage requires developing a written narrative combining participant extracts with researcher interpretation.	Participant quotes included in the written report aim to evoke resonance in readers familiar with the occupational therapy context.

Given our interpretivist philosophy, a non-foundational approach was taken to considering the indicators of quality for the research. It was also important to align these judgements with our research data and aims, but we concluded that the present work should be judged according to the extent to which it provides a substantial contribution to knowledge, methodological coherence, resonance and credibility strategies (Smith and McGannon, 2018). To evoke resonance with the often-tacit experiences of emotional labour in readers, participant quotations are presented alongside rich interpretations of the interviews by the research team. To enhance credibility, an audit trail of analytical decisions was kept in the form of electronic comments on transcripts and a codebook (Braun and Clarke, 2021). The first and second authors independently coded a selection of interview transcripts and kept reflective journals. The fifth author acted as a critical friend whereby constructive comments, questions and reflections were offered from their perspective as an experienced Occupational Therapist. Such feedback influenced the development, refinement and naming of themes as well as the overall thematic structure.

## Results

Two overarching themes captured the emotional labour experiences of occupational therapists when interacting with clients including: (a) navigating subjective emotion norms and, (b) negotiating authentic and inauthentic emotions to nurture the therapeutic relationship.

### Navigating subjective emotion norms

Participants highlighted the need to navigate a range of unwritten, contextual, and culture-bound emotion norms when interacting with clients. There was an implicit expectation to adapt emotional displays in the best interests of clients to develop the therapeutic relationship. Nevertheless, the extent to which specific emotional displays were appropriate was subjective. Two themes spoke to this complexity including (a) prioritizing client needs as a guide for desirable emotional displays, and, (b) ‘professionalism’ as a mutually protective mechanism.

#### Prioritizing client needs as a guide for desirable emotional displays

Participants emphasized the importance of prioritizing the needs of the client during a social interaction as a guide for interpreting what emotional displays were expected in a variety of situations. All participants shared that the need to actively empower clients to fulfil their occupations informed emotion norms. For example, Harley felt an implicit need to express emotions such as happiness and enthusiasm when developing rapport with clients, ‘I try to always give my clients lots of compliments . . . so I’m always trying to be super positive and friendly and motivating . . . All of OTs [Occupational Therapists] I know, pretty much everyone is the same, and they’re very like positive and motivating and like happy go lucky sort of people’. Amira echoed this sentiment, ‘It’s more socially acceptable to have your positive emotions worn on your sleeve than is your negative ones’. To remain non-judgemental and respectful towards clients, participants felt the need to suppress angry and frustrated emotional displays in response to problematic circumstances as Chuck highlighted, ‘If someone is rude to you, it might not be your first instinct to hold your tongue, but. . . we have to demonstrate good behaviour all the time’. Indeed, responding to client needs with anger or frustration was not deemed appropriate by Skye, ‘If I have more stuff going on at home like I wouldn’t wanna come into a chaotic environment because if I’m gonna yell back or snipe. . . Then it’s not gonna be professional or therapeutic really’. This implies that the participants prioritized the therapeutic relationship with clients at the expense of their own needs to uphold prevailing emotion norms.

To prioritize client welfare and promote autonomy within the therapeutic relationship, participants discussed the need to create therapeutic spaces with clients which influenced the intensity of emotional displays that were expected. For example, emotions such as sadness, crying and fear were permissible and necessary to express to an extent, especially when showing empathy to a client, but often participants avoided high-energy, unpleasant emotions to afford clients the opportunity to express themselves, ‘You can obviously have empathy and sympathy but you can’t show too much because you want to be strong for people . . . I don’t know if that’s maybe ‘cause I’m a male. I don’t want to display too much!’ (Leon). Nevertheless, some participants considered crying inappropriate as demonstrated by Cara, ‘I would not try to deter from conversations where they start crying or whatever else ‘cause I’m more confident to sit in those moments and show empathy without bursting into tears myself ‘cause I’ve got some more emotion regulation’. Conversely, Lizzy felt that being vulnerable and empathizing with clients by crying could be beneficial,

I have cried before with a patient that has told me things and it’s been sad but they like that. Sometimes I felt really unprofessional but then my boss said, ‘No, that’s good. It shows you care. You’re human. You’re allowed to do that.

#### ‘Professionalism’ as a mutually protective mechanism

When presented with challenging circumstances, participants cultivated a sense of ‘professionalism’ by suppressing emotions as a mutually protective mechanism for both themselves and their clients. Harley likened this to the ‘poker face’ approach, ‘Like covering, holding and not showing’. To protect clients in vulnerable situations, some participants shared the expectation to convey neutrality, that is, not inherently ‘positive’ or ‘negative’, towards clients. For example, when entering a client’s home, Leon discovered extremist political memorabilia, ‘Yeah, you’ve got to just be very neutral. You can’t say anything, you know?. . . It was quite concerning so I had to seek advice’. Indeed, Leon felt he could not allow this to influence the therapeutic relationship and prioritized client care by hiding their shock.

When a client’s spouse disclosed their worry about their husband passing away from dementia, Cara highlighted the importance of adopting a calm demeanour and problem-solving approach to the conversation, ‘. . .They’re not looking for an emotional response. They’re looking for someone to listen, but then providing a practical solution cause otherwise why would they have said it?’ Redirecting the focus of the therapeutic encounter onto the practitioner’s felt emotions in the moment was deemed unprofessional by this participant; therefore, it was expected that emotional displays were neutralized. Chuck recalled the need to remain neutral when entering poor residential home environments, ‘I don’t think it would be appropriate to become like visibly upset about something like that. You can voice your concern. . . but I don’t think it’s appropriate to become overly emotional . . . In any way’. Masking what could be deemed inappropriate emotions and displaying restraint was beneficial for the therapeutic relationship and reinforcing professional boundaries.

Rhia highlighted the subjective nature of interpreting what and how much emotion is appropriate to express, ‘You’re supposed to talk to everybody in a professional manner, and be empathetic and listening, but also like not overly involved. It’s tricky . . . I don’t know . . . It’s a weird one because it’s not a hard rule’. This quote highlights challenges associated with negotiating professional boundaries in light of emotion norms in the therapeutic relationship. An extension of this debate included the participants’ divergent views on the appropriateness of expressing empathy through physically touching clients. Nina highlighted the importance of context in the following quote,

I don’t really hug my patients, or hug their families. . . It’s very rare. I think I’ve found one of my patients and had a big celebratory like, ‘Oh, you’ve done it! You’re doing well! You’re going home’ . . . I just hold their hand between mine . . . I think there’s just unspoken rules about how you touch people.

From the participants’ perspective, Lizzy shared the need to remain ‘professional’ despite experiencing daily personal challenges, ‘You’re always expected to be, no matter what, always professional, it’s part of your conduct. If you’re feeling a bit in yourself, not great at times throughout things, you can’t show that’. Indeed, Delphine shared their experience of interacting with an angry client which resulted in an abrupt end to a phone call. Despite this, Delphine felt the need to express neutral and assertive emotions to control the situation, ‘’cause I think you still need to be professional. And I feel like if you show them your weak spot they are just gonna be like . . . attacking it?’

### Negotiating authentic and inauthentic emotions to nurture the therapeutic relationship

Every participant felt it necessary to enact emotional labour to comply with subjective emotion norms. The participants shared their experiences of changing how they thought, felt, and acted with clients and regardless of the type of emotional labour performed, participants grappled with the need to *appear* sincere to clients, despite the potential for surface acting or deep acting, when developing therapeutic relationships. Three themes captured the emotion negotiation process including (a) acting sincerely, (b) manufacturing desired feelings towards clients, and, (c) (in)authentic emotional expressions.

#### Acting sincerely

Participants felt compelled to suppress felt emotions deemed unhelpful towards clients such as anger, frustration or sadness by acting as sincerely as possible. Cara described this as ‘putting up a front’ when it came to regulating feelings of frustration towards patients during abusive interactions, ‘. . .Although you feel dead inside, you’re trying to still maintain that professional front. And not having a go at them . . . I guess I’m better at hiding it instead of changing it’. This illuminates the effort associated with incongruence between felt and expressed emotions in the moment when clients are abusive. Closely linked was the need to suppress feelings of frustration with clients. Lizzy recalled an interaction about prescription medication with a client with severe rheumatoid arthritis,

The condition was so bad that I felt like I wanted to say, “Just take them. You need them!” [aggressive tone]. . . But I couldn’t, I had to keep in what I thought . . . It’s hard sometimes to not be like ‘Take it, cause it’ll really help!’ [pleading tone].

Despite caring for the client, Lizzy felt unable to express their genuine concerns about their treatment and feigned a neutral demeanour. Reflecting on working in mental health wards, Millie demonstrated the importance of tempering their sadness, ‘A lot of people have got these horrendous stories and you can’t cry in front of all of them, you have to be able to regulate your emotions so you’re able to help them’.

#### Manufacturing desired feelings towards clients

Participants shared experiences of trying to purposefully change how they were feeling to express more observably sincere emotions with clients. One such technique was to rationalize problematic behaviours as Rayne regularly experienced verbal abuse from clients in forensic mental health settings yet attempted to deduce a logical explanation as to why clients were behaving in an abusive manner,

Someone might say to me like, ‘You’re an f’ing fat B I T C H’ . . . You have to regulate your emotions because I can’t turn around and say, ‘Shut the F up!’. . . I need to understand that that person is unwell and that’s the reason why they’re acting in the way they’re acting . . . In that moment, you have to maintain ultimate professionalism because you’re being hit in the most emotive part of your being.

Participants also redirected their focus away from issues that might distract them. For instance, when Michael experienced challenging personal circumstances, he attempted to manifest calmness by engaging in mindfulness exercises, ‘I should not day-dream about all these things. . . and if those things are going to interrupt me, then I try to practice being completely involved with the client’.

More physical and vocal approaches were adopted to conjure feelings as Nina verbalized mantras to shift how they were feeling before helping a client with personal care, ‘I’m very sensitive to smells, so sometimes I just literally have to be like [enthusiastic tone] ‘OK, we’re going in!. . . I’m going to do it! It’s gotta be done’. This allowed Nina to remain respectful in an intimate situation with a client. An extension of this strategy was to take deep breaths to channel appropriately intense emotions needed to interact with children as demonstrated by Rhia,

So, I would go from feeling like, ‘Argh! Oh my God. I don’t wanna do this, I don’t wanna do this . . . I can’t walk.’ And to then being like [exhale] ‘Right, I’m going to go play with a child now!’ I would be like ‘Oh my goodness! Hi!’ [high pitched, excitable tone].

#### (In)Authentic emotional expressions

Some participants argued that authentic emotional expression was possible within the confines of the therapeutic relationship without risking their sense of professionalism. Nevertheless, other participants argued that moments of authenticity were constrained by professional expectations, thus questioning the potential for authenticity as an occupational Therapist.

Some participants felt that they experienced moments of authenticity with clients when expressing happiness at their clients’ progress, ‘I’m the people’s biggest cheerleader. . . I get really excited for people when something good happens. . . I think it’s all genuine. . . I would not sit there and like fake be happy . . . I’d like to think it’d be quite obvious’ (Millie). Lizzy felt that remaining ‘herself’ helped negotiate the power dynamic with clients when making a splint, ‘. . . I’ll be like chatting away to them saying, ‘So you’re ready for Christmas?’ Although there’s like a professional front, I’m me still and I’m friendly and I want them to feel like they’re on the same level and that I’m not better’. Nevertheless, genuine emotional expressions also included moments of vulnerability with clients, as Amira described,

I think I am, on the whole, authentically emotional with patients. . . You know, if I’m sad about the situation that they’re in, I. . . will be transparent about that . . . If I’m feeling positive emotionally about something to do with work, that’s related to that patient, I will share that as well as authentically as I can.

Participants that prioritized openness and transparency within the therapeutic relationship afforded opportunities for genuine emotional expressions. Indeed, in times of crisis, Martina found it crucial to show compassion with a client experiencing suicide thoughts,

I think what we should do more of is not the prescribed kindness, but the actual kindness. So, the decisions we make or the explanations we make should come from heart in a way or from a good place. It can be less mechanical at times . . . But I think people can tell if it’s not coming from the right place.

Martina did not feel that their authentic expression of compassion affected their ability to help the client. Some participants argued that personal self-disclosure facilitated moments of genuine self-expression. For example, Cara shared that hugging or crying in response to upset clients helped set professional boundaries for appropriate emotional expressions,

They just can’t see the wood for the trees and I’ve given them a massive hug. Because it’s good to emotionally bond with people and they’re allowed to be upset. . . You have to learn to allow people to be upset and sit with them when they’re upset and show that you are upset, but then also. . . you can’t be like wailing next to them.

Despite the need to express vulnerability, Cara recognized that the intensity of emotions expressed could potentially influence sincerity and perceptions of professionalism. Participants discussed the possibility to remain authentic during all client interactions due to professional practice guidelines and workplace culture constraints,

It’s finding that middle ground between being a person, acting like a person, not like an administrative robot. . . But at the same time understanding you’re at work and it’s a job and it’s not you personally, reflecting that in this it does provide the patient a feeling of safety because they understand there are boundaries. (Cara)

The quotation above highlights the salience of setting professional boundaries that provide scope for moments of authenticity and what the practitioner would find appropriate to express.

## Discussion

This study explored the emotional labour experiences, including norms, emotion regulation and emotion performance, of qualified, practicing occupational therapists when interacting with clients. The participants found emotional labour to be an essential, subjective and complex element of nurturing the therapeutic relationship with clients. Contrary to traditional medical models that prioritize the importance of professionals acting with affective neutrality, emotional detachment and inequitable power relations during client interactions, the findings demonstrated a socio-emotional shift to therapeutic relationship management that centered client empowerment by means of emotional labour. Nevertheless, participants in this study encountered a range of emotionally taxing situations with clients that demanded emotional restraint, or expression of inauthentic emotions, to maintain professionalism.

Similar to previous research on the socialization of emotion norms in healthcare (e.g. nursing; McCloughen et al., 2020), participants shared experiences that conveyed the challenges associated with interpreting implicit, context-dependent emotion norms. A key finding in the present study was that practitioners prioritized client needs in the moment (i.e., over the course of a social interaction) to determine emotion norms with clients in light of varying practice contexts. Echoing the findings of Thomas et al. (2019), participants in this study were, at times, divergent in their approaches as to *what* and *how much* emotion was appropriate to express with clients. For example, expressing emotions such as anger and frustration with clients was deemed unacceptable (Kolehmainen and McAnuff, 2014), whereas participants were divided as to whether overt or intense displays of emotion such as crying were appropriate. Indeed, such findings extend the debate regarding whether crying enhances intentional and interpersonal relating between client and therapist (Wallengren et al., 2022) or contributes to feelings of patient guilt, worry and self-blame (Bylsma et al., 2020).

Consistent with Grandey and Gabriel’s (2015) conceptual perspective and healthcare research (Healey 2017; Riley and Weiss, 2016), participants in this study engaged in surface acting (e.g. suppressing expressions of shock) and deep acting (e.g. rationalizing problematic behaviours to understand client perspectives). Nevertheless, therapists in this study emphasized the need to be perceived as sincere by clients regardless of the emotional labour strategies adopted. For example, in emotionally demanding situations with clients involving intimate physical touch (e.g. helping with personal care), sensory overload (e.g. strong smells), and abusive behaviours (e.g. name-calling), participants felt urged to surface act and/or deep act to modify emotions that might negatively affect them and their therapeutic relationships. This implies that as long as the practitioner felt confident in their ability to express the appropriate emotion(s) that appeared sincere to clients, regardless of whether they engaged in surface acting or deep acting, this allayed concerns about the detrimental consequences of emotional dissonance when surface acting and deep acting (Hülsheger and Schewe, 2011). Indeed, participants also shared moments of authentic emotional expression with clients, yet acknowledged the challenges with expressing genuineness within the constraints of professional boundaries (Riley and Weiss, 2016).

The results of this study offer several implications for the professional development, support and supervision of occupational therapists in relation to emotional labour demands. Specifically, the findings can be interpreted to raise questions about whether emotional labour represents a socio-emotional capability for occupational therapists due to the need to enact emotional labour in response to challenges, varying contexts and to achieve objectives with clients. Indeed, perceptions of professional competence in medicine have been associated with emotional composure (Crowe and Brugha, 2018). At present, emotional labour does not feature in professional codes of conduct that govern how occupational therapists are expected to behave when interacting with clients (Royal College of Occupational Therapy, 2021; World Federation of Occupational Therapists, 2016). Therefore, theoretical and applied discussions of emotional labour demands as an occupational therapist might not feature in university and/or professional development curricula. Future research could focus on creating evidence-based training materials to enhance occupational therapists’ awareness and preparedness for emotional labour demands by providing career-long opportunities for therapists to practice, receive feedback on and finetune emotional labour skills. Co-production approaches involving therapists, educators and service users could be used to develop fit-for-purpose materials that expose trainees to challenging social interactions within therapeutic relationships.

The present findings suggest the need for therapists to explicitly reflect on their emotional labour, given its vital role in building therapeutic relationships. This process would facilitate critical reflection on the influence of socio-political practice contexts on emotional labour demands, help define and challenge emotion norms and reduce stigma associated with discussing emotional demands, ultimately enhancing quality of client care (Guy et al., 2020). With specialist training to support supervisors and managers, such reflections could be facilitated via one-to-one or group supervision whereby guidance, feedback and resources to help practitioners recognize and manage emotional demands effectively could be provided (Martin et al., 2022). Through regular supervision sessions, practitioners can gain insights as to when there is emotional incongruence as well as learn coping strategies to help navigate emotionally demanding situations (Horton et al., 2022). Reflective practices and supervision could help practitioners make implicit understandings of emotions explicit, enhancing their self-awareness and readiness for emotional labour demands.

While the cross-sectional research design of the present study permitted participants to share their stories about emotional labour with clients, a single interview did not lend itself to understanding how emotional labour strategies might change over time or throughout the course of social interactions with clients. Nor did the research design allow us to attend to the potential influence of social identities (e.g. race/ethnicity, gender, sexuality, religion) and organizational structures on emotional labour, nor the medium-to-long-term effects of emotional labour on the wellbeing of therapists. In addition, the participants recruited represented a range of working contexts and field experience which might have diluted practice-setting specific learnings about emotional labour. Therefore, future research could adopt mixed-methods longitudinal designs (e.g. ethnographical observations, experience sampling) that analyse the relationships between emotional labour and subjective wellbeing, job satisfaction, occupational health, burnout and turnover intention in specific disciplinary contexts (e.g. paediatric unit vs frailty unit; Rogers et al., 2014). Such research would afford methodologically robust practice-based insights for trainee and qualified practitioners (Hings et al., 2024).

In conclusion, emotional labour represents a prevalent, variable and challenging demand for occupational therapists to negotiate when interacting with clients. The participants in this study prioritized the need to be perceived as sincere by clients, regardless of the emotional labour strategies adopted, amid ongoing personal and professional circumstances. The findings contribute to bridging the emotional labour knowledge-practice gap (Hings et al., 2024) in terms of the emotion-related knowledge, skills and abilities required for good quality person-centred healthcare in occupational therapy. Future research could progress knowledge of emotional labour including discipline-specific exploratory investigations, the development of educational interventions designed to enhance awareness and preparedness for emotional labour with clients, and comprehensive investigations linking emotional labour to a range of client, occupational, wellbeing outcomes.

**Key findings**

- Emotional labour is a practice capability required to nurture the therapeutic relationship with clients.
- Therapists prioritized perceptions of emotional sincerity by clients regardless of the emotional labour strategies adopted.

**What the study has added**

- This study provides a novel extension of emotional labour theory by highlighting the subjectivity and challenges experienced by occupational therapists when negotiating emotion norms and expressions within the therapeutic relationship.
